# Gastric Adenocarcinoma with Yolk Sac Tumor Differentiation and Liver Metastasis of Yolk Sac Tumor Component

**DOI:** 10.1155/2013/923596

**Published:** 2013-11-02

**Authors:** Chhagan Bihari, Archana Rastogi, K. N. Chandan, Vikas Yadav, Dipanjan Panda

**Affiliations:** ^1^Department of Pathology, Institute of Liver and Biliary Sciences, D-1, Vasant Kunj, New Delhi 110070, India; ^2^Department of Hepatology, Institute of Liver and Biliary Sciences, D-1, Vasant Kunj, New Delhi 110070, India; ^3^Department of Oncology, Institute of Liver and Biliary Sciences, D-1, Vasant Kunj, New Delhi 110070, India

## Abstract

Gastric adenocarcinoma with yolk sac tumor (YST) differentiation has rarely been reported. We report a case of primary gastric adenocarcinoma with yolk sac tumor differentiation and liver metastases of the YST component in a 50-years-old patient. This was suspected due to high serum level of alpha fetoprotein in the presence of a gastric fundal tumor. Gastric carcinoma with yolk sac tumor component is a rare entity with a poor prognostic outcome.

## 1. Introduction

Yolk sac tumors (YST) are usually germ cell tumors, which arise in the gonads as primary tumors or as part of mixed germ cell tumors. They also occur in extragonadal and extrauterine sites particularly at the midline, such as mediastinum, retroperitoneum, and in the central nervous system [1]. This tumor also has been reported as a component in carcinomas with heterogeneous differentiation in the lung, stomach, large intestine, gallbladder, pancreas, and urinary bladder [2]. Yolk sac tumor as a dedifferentiated component of gastric adenocarcinoma has rarely been reported. There are only a few case reports in the published literature. Here, we present a case of gastric adenocarcinoma with yolk sac differentiation and the liver metastasis of yolk sac tumor component.

## 2. Case Report

**Figure 1:**
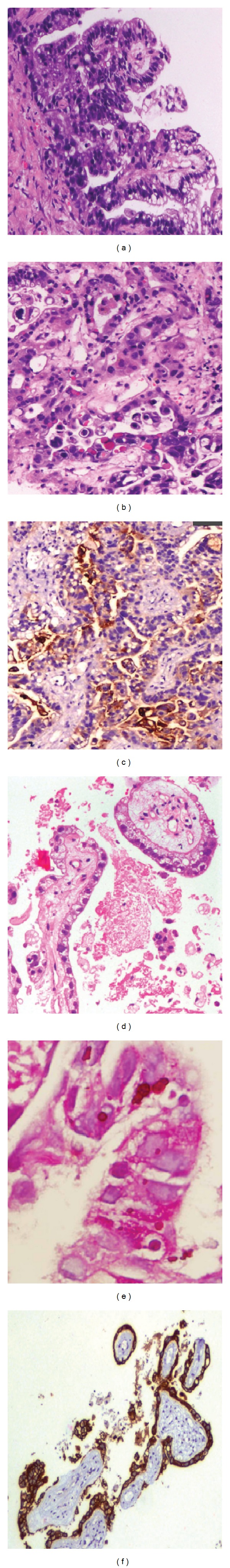
(a) Gastric adenocarcinoma papillary growth pattern. (b) Gastric tumor showing yolk sac tumor (YST) area with hyaline globules. (c) Gastric YST glypican 3 positive. (d) Liver YST metastasis showing papillaroid fragments. (e) Liver metastasis of YST showing cytoplasmic PAS-positive, diastage-resistant hyaline globules (400X). (f) Liver YST papillary fragment showing glypican 3 positivity.

A 50-year-old male presented with complaints of pain in the abdomen and weight loss for one and half months. On ultrasonography examination, he was found to have multiple liver space occupying lesions. Upper gastrointestinal endoscopy revealed an ulcerated growth in the stomach at the fundus region. The laboratory findings showed high serum level of alpha fetoprotein levels 2291 ng/mL (normal < 5 ng/mL) and raised carcinoembryonic antigen (408 ng/mL, normal < 5 ng/mL). The biopsy was done from the gastric growth that showed adenocarcinoma with areas of yolk sac differentiation in few areas of the biopsy fragments. Yolk sac tumor component showed broad papillary and festooned architecture lined by atypical stratified epithelial cells with enlarged, hyperchromatic nuclei showing high nucleocytoplasmic ratio, irregular nuclear contours, coarse chromatin, prominent nucleoli, and abundant vacuolated cytoplasm with occasional hyaline globules (Figures 1(a) and 1(b)). Immunohistochemically, the yolk sac tumor component was positive for low molecular weight cytokeratins (CK-8 and CK-18), CD30, placental alkaline phosphatase (PLAP), alphafetoprotein (AFP) and glypican 3 (Figure 1(c)). Tru-cut biopsies were also taken from the liver lesions under ultrasound guidance, considering the high serum levels of AFP in order to rule out the possibility of coexisting hepatocellular carcinoma (HCC). Liver biopsy from the liver lesions also displayed only YST comprising papillary and festooning architecture with intracytoplasmic and extracytoplasmic hyaline globules (Figures 1(d) and 1(e)). The metastatic tumor was also positive for CK8, CK18, PLAP, AFP and glypican 3 (Figure 1(f)). Histopathological diagnosis of gastric adenocarcinoma with yolk sac tumor differentiation with metastatic yolk sac tumor was rendered. Patient was briefed about the poor outcome of the disease and given choice to opt chemotherapy for palliation, for which he declined. 

## 3. Discussion

Gastric yolk sac tumor is a rare entity. There are only twelve case reports of gastric yolk sac tumor (YST), most of them associated with gastric adenocarcinoma and considered to be the yolk sac tumor differentiation of primary gastric adenocarcinoma [3]. Pure YST of stomach is a very rare entity; only two cases have been reported in the English literature [3]. This type of tumor is usually seen in elderly patients with advanced metastatic disease. Lymph node, lung, and liver metastasis had been detected at the time of presentation or during autopsy examination [3]. Histopathologically, the yolk sac tumor is identified by presence of variable architectural pattern, including reticular, microcystic, macrocystic, papillary, and characteristic Schiller-Duval bodies. The tumor cells frequently show intracytoplasmic vacuoles or PAS-positive, diastage-resistant hyaline globules [4, 5]. Yolk sac tumor cells produce immunoreactivity AFP, so the serum AFP levels are generally high, and AFP can be demonstrated immunohistochemically within the tumor [5]. Immunohistochemical panel for YST diagnosis is a combination of low molecular weight cytokeratin (CK-8, CK-18), AE1/AE3, glypican 3, CD117, CD30, AFP, PLAP, and OCT3/4, which distinguishes YST from other neoplasms [6]. Our patient had gastric tumor with liver metastasis and raised serum AFP level. Biopsy examination from both sites showed YST tumor with papillary growth pattern and intracytoplasmic and extracytoplasmic hyaline globules. The tumor cells were CK8, CK18, CD30, glypican 3, and PLAP positive. 

The pathogenesis of germ cell tumors arising in non-gonadal organs, such as the stomach, is not exactly known; however, it is considered that it may be adenocarcinoma heterologously transformed or progressed to yolk sac tumor components through retrodifferentiation or alternate differentiation pathway [3, 4]. Recently, it is considered that these tumors arise from the tumor stem cells present in the somatic tumor [7]. 

Gastric adenocarcinomas with YST elements are very aggressive tumors which usually present with lymph node, lung, or liver metastases, and consequently, the prognosis is generally poor. In the small gastric or liver biopsy tissue, YST should be suspected when there are raised serum AFP levels in absence of gonadal tumor and hepatocellular carcinoma.

## References

[1] Puglisi F, Damante G, Pizzolitto S (1999). Combined yolk sac tumor and adenocarcinoma in a gastric stump: molecular evidence of clonality. *Cancer*.

[2] Satake N, Chikakiyo M, Yagi T, Suzuki Y, Hirose T (2011). Gastric cancer with choriocarcinoma and yolk sac tumor components: case report. *Pathology International*.

[3] Kim YS, Kim SH, Seong JK, Lee BS, Jeong HY, Song KS (2009). Gastric yolk sac tumor: a case report and review of the literature. *Korean Journal of Internal Medicine*.

[4] Garcia RL, Ghali VS (1985). Gastric choriocarcinoma and yolk sac tumor in a man: observations about its possible origin. *Human Pathology*.

[5] Wang L, Tabbarah HJ, Gulati P, Rice S, French SW (2000). Gastric adenocarcinoma with a yolk sac component: a case report and review of the literature. *Journal of Clinical Gastroenterology*.

[6] Kao C-S, Idrees MT, Young RH, Ulbright TM (2012). Solid pattern yolk sac tumor: a morphologic and immunohistochemical study of 52 cases. *American Journal of Surgical Pathology*.

[7] Nogales FF, Preda O, Nicolae A (2012). Yolk sac tumours revisited. A review of their many faces and names. *Histopathology*.

